# The Japanese Educational System as an International Model for Urban Resilience

**DOI:** 10.3390/ijerph18115794

**Published:** 2021-05-28

**Authors:** Elisa Gavari-Starkie, María-Francisca Casado-Claro, Inmaculada Navarro-González

**Affiliations:** 1 Departmento de Historia de la Educación y Educación Comparada, UNED, 28040 Madrid, Spain; egavari@edu.uned.es; 2 Department of Economics and Business, Universidad Europea, 28670 Madrid, Spain; francisca.casado@universidadeuropea.es; 3 Departmento de Historia de la Educación y Educación Comparada, UNED Centro Asociado de Albacete, 02007 Albacete, Spain

**Keywords:** disaster risk reduction, disaster resilience education, urban resilience, sustainability, Japan, bosai culture, disaster preparedness

## Abstract

Global cities in the context of accelerated urbanization have to deal with more diverse risk factors than ever before, which highlights the need for a faster and more creative response capacity. Although it is necessary to strengthen technical systems, since they are surrounded by human systems, individual resilience will help to strengthen the community. The educational system is key to developing the human factor in a world where various systems in global cities are increasingly interconnected, which in turn increases risks. Japan is fostering a culture of disaster risk reduction in both the formal, non-formal, and informal education sectors, in which creativity and autonomy are key competencies. Tokyo is the highest populated metropolitan area globally, and its educational system is the international model for education in disaster risk reduction. Urban areas around the world face similar challenges and experience similar needs. This article addresses the challenges that the human factor faces in large cities and the possibilities of increasing resilience in both individuals and communities through Disaster Resilience Education (DRE), taking the Japanese educational system as a model.

## 1. Introduction

The main goal of education for disaster preparedness, formally called Disaster Education, consists in cultivating from an early age the knowledge and skills needed to take action in case a disaster arises in order to be able to act proactively and reduce the consequences. This ability to respond appropriately to disasters entails both knowledge (theory) and practical training (drills), to develop skills that are closely related to other soft skills (critical thinking, problem-solving, or teamwork, amongst others). Altogether, they can be lifesaving during and after an emergency. Nevertheless, most educational systems focus on knowledge acquisition and hard skills rather than developing life-sustaining skills and practical information that can protect individuals and communities in an emergency by building resilience into the system.

Even though few educational systems include Disaster Education in their curricula, “cultivating the ability to live” is a skill fostered throughout the Japanese educational system with specific activities aimed at each educational level, both in formal curricular subjects and extracurricular activities. The authors of this article contend that Japan has gone one step further by building into the national school curriculum the elements needed to foster resilience in both individuals and communities, hence becoming a role model for other countries, cities, and municipalities worldwide. The Japanese Disaster Resilience Education (DRE) model focuses not only on learning about natural hazards in the local environment and how to stay safe before, during, and after an emergency or disaster but also on making connections between schools and their surrounding communities. 

In October 2020, the UNDRR (United Nations Office for Disaster Risk Reduction) launched its initiative Making Cities Resilient, MCR 2030 [1], which started operating in January 2021 and will be active until December 2030, aiming to make cities inclusive, safe, resilient, and sustainable, as two-thirds of humanity will be living in urban areas by 2030. The MCR 2030 initiative is closely linked to other United Nations frameworks with a horizon 2030, as is the case of the Paris Agreement for Climate Change, the Sendai Framework for Disaster Risk Reduction, and the New Urban Agenda, as well as the Global Development Goals, and in particular SDG 11: Sustainable cities and communities. 

Since they concentrate economic and political power, as well as increasing populations, cities and urban areas will play an important role in the global stage, which comes with a series of challenges and responsibilities. The COVID-19 pandemic has brought to the fore some of them, as the crisis affected urban areas where population density is higher in contrast to other areas with more sparse populations. One could even say that the virus traveled from city to city, entering countries through international airports similar to an accidental tourist and then moving from urban areas to rural ones. In big cities, businesspeople and health workers were the first to come into contact with and get infected by the virus; then, it spread through public transportation where working-class citizens got infected. 

The crisis has underlined problems that were already present, such as social inequality or the economic gap between the upper classes and the working classes, impacting the health systems of both developed and developing countries. All this highlights the need to strengthen physical infrastructures and the human systems surrounding them to build a more sustainable future. Education plays a central role in strengthening human systems and, hence, building resilience in urban areas and beyond. 

Policymakers should bear in mind that resilience starts with education. It is imperative on the one hand to foster soft skills and, on the other hand, to insert disaster preparedness, sustainable development, and environment-related subjects in formal education (main subjects in school curricula), non-formal education (complementary subjects in curricula), and informal education (outside the classroom setting). Since today’s children will be the citizens of tomorrow, the education they receive will determine how they will envision global challenges such a climate change. Moreover, the skills they develop should enable them to navigate rough seas and be prepared to act if an emergency arises. 

Whereas the role that education plays in raising disaster awareness amongst students, teachers, and parents, as well as in building more resilient communities, is widely recognized, the truth is that Disaster Education is not a broadly spread practice around the world yet. Even in countries where its importance is recognized, there is still room for improvement, because in some cases, it does not encompass all types of disasters, while in other cases, it focuses on post-disaster recovery, or it is not part of formal education, and instead, it is considered a complementary extracurricular activity to be carried out in a non-formal setting. Shiwaku and Fernandez [2] contend that “in many parts of the world, there is no separate or special curriculum for disaster education in elementary and high schools”. Gwee, Shaw, and Takeuchi [3] analyze the case of Iran, where disaster education is carried out through formal and non-formal educational activities in the *Earthquake Safety Education Program*, which revolves around the most common type of natural disaster in the country.

Examples of Disaster Education can be found in both developed and developing countries worldwide, from Australia to the United States, from South East Asia to Latin America, Africa, and the Caribbean. However, amongst them, few educational systems follow a systematic implementation of education for disaster preparedness in their national curriculum, which stems from governmental policies. Therefore, owing both to its breadth and its depth, the Japanese educational system can be considered an example of best practices in the field of DRE for various reasons [4]. To start with, DRE is part of formal, non-formal, and informal education. Secondly, it focuses on various types of disasters (both natural and human-made). Thirdly, it boasts strong governance through its integration in the national, regional, and local context. Fourthly, it follows an all-of-society approach reaching out to communities beyond school and promoting active participation of various stakeholders (students, mothers and fathers, teachers, neighbors and civil society organizations, such as *jishu bosai soshiki*, i.e., autonomous organizations for disaster risk reduction).

Moreover, following the traditional Japanese philosophy of continuous improvement (kaizen) and attention to detail, as well as their penchant for extracting positive lessons from adverse events, throughout the years, the Japanese Disaster Risk Reduction (DRR) model has become increasingly sophisticated. Not in vain, Japan works closely with the UNDRR, has organized three UN World Conferences on Disaster Risk Reduction, and is eager to share its experience and know-how with the rest of the world, becoming a beacon in this field. 

This article analyzes the Japanese educational system as a role model in fostering resilience in individuals and communities from a perspective of DRR and education. As a disaster-prone country, Japan has developed theoretical and practical guidelines that have been incorporated into the educational system to make students ready to act proactively in adverse situations, such as when a natural disaster occurs. DRE is more often than not neglected in most educational systems, even though it fosters skills that could be lifesaving in an emergency. Therefore, this article aims to outline how educational systems can foster resilience in urban areas by focusing on the case of Japan and the lessons that can be extracted from the country’s long tradition in disaster risk management and reduction. 

Using a descriptive and analytical research methodology, this article proceeds in three parts. In the first part, the framework for analysis of Disaster Resilience Education (DRE) is introduced, and the leading role of Japan in DRR is revised. In the second part, education is presented as a valuable tool in resilience development, before examining the reforms in the Japanese educational system leading to the incorporation of DRE into the curriculum. Finally, the lessons that can be transferred to urban resilience are analyzed in the discussion section, and some conclusions are extracted. 

## 2. What Is DRE and Why Is It Important for Urban Resilience? 

About half the global population lives in urban areas as of 2021. By 2030, this figure is expected to increase to 60% [5]. Cities are already quite complex habitats, but their complexities will be multiplied as their populations grow, exacerbating some of their problems: inequality, air quality, health issues, unemployment, overcrowding, etc. On the other hand, the complex relationship between urban areas and risk is more present in the face of natural hazards and human-made ones, leading to disasters whose consequences can potentially affect a large population. Moreover, Sanchez et al. [6] point out that cities worldwide seek to improve their resilience to face the challenges posed by climate change (sea-level rise, heatwaves and droughts, disrupted rainfall patterns, amongst others). 

We start this article by setting the topic in context by defining resilience and urban resilience. Then, we explore some global initiatives to build urban resilience before explaining why DRE is important for urban resilience and why Japan is a model in disaster education. 

### 2.1. What Is Resilience?

Broadly speaking, resilience could be defined as the ability to face difficulties adequately. This capacity is conditioned by the strengths and weaknesses of an individual or within a given community or society. The term ‘resilience’, which originated in ecology, was first defined in 1973 by Holling [7] as a measure of the persistence of ecosystems and their ability to absorb change and return to their normal stability or equilibrium after a temporary disturbance. According to some authors, resilience is the ability of a system to quickly recover its original state (to bounce back, to jump back, to rehabilitate, or to recover), while for the US Global Resilience Institute (GRI), it is the ability to withstand, recover, and adapt to periodic shocks and major shocks. 

The UNDRR defines the term resilience as “the ability of a system, community or society exposed to hazards to resist, absorb, accommodate, adapt to, transform and recover from the effects of a hazard in a timely and efficient manner, including through the preservation and restoration of its essential basic structures and functions through risk management” [8]. The term derives from the Latin words *resilire* (to bounce back) and *stare* (to resist), which transmit the double-faceted nature of the concept in which dynamic and static stances converge. On the one hand, resilience is about being able to resist in the face of adversity (stare); while, on the other hand, it is the ability of a system to return to its original state after a shock or, if that failed, to accommodate or adapt to the new situation instead. 

### 2.2. What Is Urban Resilience?

According to the Global Resilient Cities Network (Table 1), urban resilience is “the capacity of individuals, communities, institutions, businesses, and systems within a city to survive, adapt, and grow no matter what kinds of chronic stresses and acute shocks they experience” [9].

However, Sanchez et al. [6] (pp. 2–3) contend that urban resilience is a contested concept as there is not a single agreed definition for the term. The existing definitions range from short-term to long-term and from a narrow focus to encompassing various aspects and potential threats. After systematically revising 82 documents on the subject published in English from 1970 to 2016, the authors classified them into eight categories summarized in Table 1. 

### 2.3. Global Initiatives to Build Urban Resilience

The need for urban resilience is not new; it is a pursuit that has been around for years, but the urgency to act before it is too late is ever more impending. Climate change has given new impetus that will be more detrimental in cities because not only do they have high population densities, but they are economic and political centers of activity as well. 

Several global initiatives aim to build urban resilience, following different programs and with a varied membership. A selection of initiatives is explored in the following lines: the UNESCO Global Network of Learning Cities, the MCR2030, the Global Resilient Cities Network, the United for Smart Sustainable Cities, and the C40. 

Firstly, the UNESCO Global Network of Learning Cities is an international policy-oriented network acting as a hub where cities can interact and exchange ideas. It also provides technical assistance and support to SDG 4 (‘Ensure inclusive and equitable quality education and promote lifelong learning opportunities for all’), and SDG 11 (‘Make cities and human settlements inclusive, safe, resilient, and sustainable’) is at the core of its endeavors [10]. 

Secondly, the MCR2030 (Making Cities Resilient) is a UNDRR initiative that seeks to make cities more inclusive, safe, resilient, and sustainable by 2030. Launched in October 2020, the program started operating in January 2021 [1]. 

Thirdly, the Global Resilient Cities Network (R-Cities), which saw the light in 2020, is a spin-off of the previous Rockefeller Foundation 100 Resilient Cities, which is an organization created in 2013 that gained both recognition and respect on the global stage. Under this program, cities appoint Chief Resilience Officers who are in charge of developing a resilience strategy and plan resilience-guided actions that have a positive impact in the cities [9].

Fourthly, the United for Smart Sustainable Cities (U4SSC) initiative of the UNECE (United Nations Economic Commission for Europe) pursues two objectives: (1) generating guidelines, policies, and frameworks to integrate ICTs into urban operations, and (2) helping streamline smart, sustainable city action plans with achievable targets [11].

Finally, the C40 consists of a group of 40 cities committed to the Paris Agreement for Climate Change that are taking climate action to the next level. Amongst their initiatives, offering services and organizing networks around various concerns: Air quality; Energy and buildings; Adaptation implementation; Food, waste, and water; Transportation and urban planning; and Adaptation implementation [12].

### 2.4. The Role of DRE in Urban Resilience. The Case of Japan

Since the term “urban” is understood as a system made up of ecological, social, and technical components, human and social factors play a vital role in urban resilience. Some practical approaches in our literature review emphasize the need to strengthen infrastructure and buildings (engineering and built-in resilience), leaving the human factor behind. Social resilience, defined as “the capacity of a community or society to cope with, and adapt to, disturbances or changes” [13], encompasses the ability to respond proactively to sudden changes by absorbing disturbances, self-organizing, learning and adapting. Moreover, since governments must protect their citizens, resilience can be regarded as a public good. Therefore, it should be both promoted by government policies and publicly funded.

The second Fundamental Plan for National Resilience (FPNR) of the Japanese Government, which came into force in 2019, revolves around four key areas: infrastructures, institutions, economy, and society. The FPNR sets Japan as one of the countries that are leading the promotion of national resilience-building by taking the initiative in implementing the priorities of action of the Sendai Framework for Disaster Risk Reduction. According to DeWit et al. [14], the plan identifies 179 key performance indicators (KPIs) and was funded at roughly 5 trillion yen per year since FY 2018. One of the KPIs is the share of schools that educate in disaster safety, which in 2015 stood at 99.7%, according to the MEXT [15]. 

The FPNR, which has its initial focus on large-scale natural disasters, considers fundamental combining structural measures (such as developing disaster management facilities) with non-structural measures (such as emergency drills and disaster management education) [15] (p. 4). It also establishes 12 sectors of measures concerning National Resilience, amongst which “disaster management education”, which promotes disaster prevention exercises and disaster prevention education through schools, workplaces, local autonomous organizations, and other relevant organizations. Thus, schools continually organize disaster prevention exercises (safe evacuation routes in case of an earthquake, hazard maps, and guidance signs for designated emergency evacuation sites [15] (p. 73). Disaster management is considered systemically, i.e., through interactive communication between the different social agents with educational, training, and information tools to teach how to act autonomously and follow one’s own criteria.

In order to make cities more livable, policies will have to be implemented to make them more sustainable from a social, environmental, and economic perspective, but cities also need to be inclusive, safe, and resilient. Education will play a central role in making this transition toward a safer, more sustainable, more inclusive, and more resilient urban model. Education is one of the various building blocks contributing to fostering urban resilience. Through DRE, human beings are taught to be prepared to face disasters and play a proactive role in post-disaster recovery. 

Therefore, education should carry the banner for the configuration of the cities of the future built with sustainability and resilience in mind. The education of future generations should be prioritized if we want to respond to the challenges that climate change and the exponential growth of urban areas pose for 21st-century society. The Japanese case is an example with DRR programs that are also supported by a strong civil society that is aware of the need for skills in creativity and innovation, which can face the challenges of the present and the near future, and form proactive and resilient communities. 

Japan’s capital, Tokyo, has been positioned since the 18th century as one of the most urbanized cities in the world and a referent in terms of global cities, alongside New York and London [16]. This is important to frame the current process of creating some thirty mega-cities where more than half the world population is expected to live by 2050. An accelerated process of urbanization, which is completed with more than 500 urban agglomerations featuring more than a million inhabitants.

The convergence of natural disasters and subsequent recovery positions Japan as a paradigm of urban resilience. Disasters have provided an opportunity to build back better by modifying urban planning, as well as rationalizing and modernizing the country. As the historian Yoshimi Shunya [17] points out, destruction is the most important driver in the evolution of Tokyo. The destruction caused by either conflict (such as the US bombing of Tokyo in 1945 during the Second World War) or, in most cases, by natural catastrophes (such as the 1923 Tokyo Earthquake). Japan has managed to recover and learn from adversity, whereas Tokyo can be considered an outstanding example of urban resilience. Even if its incorporation arrived later, education is vocal in building resilience at a national, regional, and local level. This article illustrates how the Japanese educational system has contributed to building resilience in individuals and communities, both in rural areas and cities. 

## 3. Why Is Japan a Model for Disaster Risk Reduction?

As a result of its geographical location in the so-called Pacific Ring of Fire, the archipelago of Japan has been frequently battered by natural disasters. Making a virtue out of necessity, the Japanese have extracted valuable lessons, which have resulted in a long tradition in disaster management. Those lessons reflect the wisdom of the people of Japan in the subject and integrate a culture of its own: the DRR culture or *bosai* culture. A part of which is informal and reflected in various proverbs and tales, whereas another part can be found not only in the scientific and academic debate (research agenda) on urban resilience, but also in the political and social agendas, where the *Sendai Framework for Disaster Risk Reduction* occupies a prominent position. These initiatives are discussed in the paragraphs that follow. 

### 3.1. Bosai Culture. A Universal Japanese Philosophy of Disaster Risk Reduction

“Bosai” is a traditional Japanese term, which involves a holistic approach to reducing the consequences of disasters (deaths, economic losses, damage to infrastructure, among others) through activities in all phases of the disaster: response, recovery, mitigation, and, above all, prevention. The *bosai culture* or DRR culture encompasses a series of behaviors and practices developed in communities that are often affected by disasters, with each community adopting its own rules that are transmitted from generation to generation. In Japan, disaster preparedness is approached from three levels: public support, community support, and self-help [18].

Being a country prone to natural disasters (earthquakes, tsunamis, torrential rains, typhoons), Japan has made a virtue of necessity as circumstances have forced it to adopt measures to mitigate the effects of disasters and prevent risks. Throughout its history, the country has been incorporating into its culture all that learning based on the experience acquired dealing with adversity disaster after disaster, to which continuous technical innovations, designed to face disasters with the greatest guarantees of reducing their repercussions, have been added. Through its close collaboration with the UNDRR (UN Office for Disaster Risk Reduction) and its official development aid policy, Japan has sought to be a benchmark on the international scene in terms of DRR. Furthermore, all its DRR actions are aligned with the Sustainable Development Goals and climate action.

Since the 1980s, Japan has been regarded as an expert country in disaster prevention and reduction at an international level and has established itself as an exporter of dialogue and reflection for Disaster Risk Reduction (DRR), promoting international cooperation actions in this matter through two salient lines of actions: (a) the organization of three United Nations World Conferences on Disaster Risk Reduction in its territory, which will be explored in the following section of this article; and (b) its official development aid (ODA) policy, which is based on the concept of human security, is managed by the Japan International Cooperation Agency (JICA), and is channeled through its official development assistance programs on DRR [19]. This international cooperation is not only about economic aid, as it also entails technology transfers and hands-on in the field training by Japanese experts in various local projects. 

### 3.2. The Significance of Resilience in Disaster Risk Management and Reduction 

One of the most notable actions of Japan in the area of risk and disasters is the organization under the auspices of the UNDRR of three World Conferences on Disaster Risk Reduction that have produced three seminal documents, which are intended to be referents in DRR policies for local, regional, and national administrations. Japan has supported these initiatives in its effort to get a better understanding of the aspects that could be improved to reduce the effects of disasters and pass the lessons on to the international community. However, these World Conferences are also a way to commemorate disasters themselves. Part of the Japanese *bosai* culture is keeping alive the memories of disasters, the loss of lives and livelihoods, as well as the material and psychological damage that they entail. It is essential not to forget; memorials are a reminder that everyone has to stay alert because disasters happen when people forget about them (*saigai wa wasureta koro ni yatte kuru*), as an old Japanese saying states [20]. 

Around the fourth anniversary of the Great East Japan Earthquake, the Third World Conference on Disaster Risk Reduction of the United Nations was held in Sendai City, from which a valuable working document emerged: the *Sendai Framework for Disaster Risk Reduction 2015–2030* [21]. Both the choice of the date and the location had a great symbolic weight: on March 11, 2011, the region was struck by the largest earthquake registered in the archipelago since records are kept, its epicenter was located off the coast of the Tohoku, the region in the which the city of Sendai is located. The earthquake triggered a series of tsunami that unchained an accident at the nuclear power plant of Fukushima I. The combination of two natural disasters (earthquake and tsunami) and a technological accident has earned it the name of Triple Disaster. 

The two previous World Conferences on Disaster Risk Reduction have also been held on Japanese soil: the first one in Yokohama in 1994 produced the *Yokohama Strategy and Plan of Action for a Safer World* [22]; the second in the city of Kobe (Hyogo Prefecture) in January 2005, commemorating the tenth anniversary of the Great Hanshin-Awaji Earthquake, better known as the 1995 Kobe Earthquake, in which the *Hyogo Framework for Action 2005–2015* was defined [23]. 

Each one of the conferences contributed to the development of the doctrines of DRR, some of which are summarized in Table 2. For the purpose of this article, the authors would like to single out the fact that the 2005 *Hyogo Framework for Action* considered that education is at the heart of prevention and, therefore, education holds the key to developing an authentic culture of DRR. In this sense, the need to use knowledge, innovation, and education to build resilience through prevention was recognized at the conference. Moreover, as this was the conference that made the concept of “resilience” go mainstream, the references to resilience are abundant in the *Hyogo Framework for Action*. Point 2 of the preamble of the text focuses on “increasing resilience to disasters with a renewed sense of urgency in the context of sustainable development (…) and integrating, as appropriate, both disaster risk reduction and increased resilience in policies, plans, programs (…)”. As a matter of fact, the resolution approved by the General Assembly on 3 June 2015, includes the term resilience as many as 35 times [24]. 

### 3.3. The Sendai Framework for Disaster Risk Reduction: 2015–2030

As for the *Sendai Framework,* although it was the first of a series of instruments adopted by UN members between 2015 and 2016, it has not gained as much fame as some of its companions. These instruments have two things in common: the same date on the horizon (2030) and some interconnected goals (sustainable development, environmental care, and human well-being, amongst others). Therefore, they are interdependent, and achieving the goals of one will determine the extent to which the goals of the rest can be attained. The two most prominent instruments in the pack are the Sustainable Development Goals (replacing the Millennium Development Goals) and the Paris Agreement for Climate Change (the latest arrangement in the UN Framework Convention on Climate Change, its predecessor was the Kyoto Protocol). Without their popularity, but still important and related to the subject of this article, the New Urban Agenda aims at creating resilient cities and communities. Finally, the Agenda for Humanity pursues, amongst others, the eradication of poverty.

Unlike the previous *Hyogo Framework for Action*, which focused on disaster management, the *Sendai Framework* focuses on ‘risk management’, and similar to the other above-mentioned international instruments, it has a broad and inclusive all-of-society approach. In other words, the responsibility for DRR rests not only on governments, whose leadership is undoubtedly essential, but also on “the public and private sectors and civil society organizations, as well as the academic community and scientific and research institutions”. In addition, vulnerable groups must take part in its design and application, including women, children, the youth, the elderly, people with disabilities, the poor, migrants, indigenous peoples, with the support of volunteers and the community [21], Preamble, Art [7].

The *Sendai Framework* is a synthetic document that defines the following: four priorities for action; seven clearly delineated objectives; and thirteen guiding principles. On the one hand, its application, at the local, regional, national, and global levels, is a call for action to all interested parties, and international cooperation is needed. On the other hand, the application of the *Sendai Framework* is supported by the Global Platform for Disaster Risk Reduction and regional platforms (African-Arab, the Americas and the Caribbean, Asia, and Europe), which are mechanisms that ensure coherence between the agendas, and are in charge of monitoring and periodic revisions, providing support to the UN governance bodies.

## 4. Education as a Tool for the Development of Resilience in the Japanese System

The central role played by the human factor in urban resilience cannot go unnoticed. The case of the Japanese educational system is an excellent example. Kitagawa [25] cites three framework laws that underpin Disaster Risk Education: the *Disaster Countermeasures Basic Act*, the *Basic Act on Education*, and the *School Health and Safety Act*.

The first one, the *Disaster Countermeasures Basic Act*, requires public organizations to draft Disaster Management Operational Plans that define actions locally. The law states that the Ministry of Education, Culture, Sports, Science and Technology (MEXT) is responsible for the following tasks: (a) organizing meetings of experts to advise on the creation of policies on disaster education, (b) making decisions on the inclusion of disaster education in the curriculum, (c) offering guidance and developing educational materials, and (d) providing teachers with leadership training on disaster awareness and skills.

The second one, the *Basic Act on Education*, establishes the school curriculum for which MEXT is responsible. This law includes the revision of study plans, a task undertaken every 10 years in Japan, whereas the implementation of each revision takes place a few years after the revision to allow time for preparation. DRE has not been defined as an independent subject in the curricula, although the content has been the object of intense debate after the 2011 Triple Disaster.

The third law is the *School Health and Safety Act*, which instructs a political framework called “School Safety” that schools develop throughout all of their activities under the guidance of MEXT. The Comprehensive School Safety Framework, endorsed through the Global Alliance for Disaster Risk Reduction and Resilience in the Education Sector (GADRRRES), encourages schools to develop a *School Safety Plan* structured in three main pillars: (a) safe learning facilities, (b) school disaster management, and (c) risk reduction assessment and resilience education [26].

### 4.1. The Japanese Educational System

Established after the Second World War, the Japanese educational system has remained practically unchanged, following the same organization all along: namely, (6+3+3+2/4) representing six years of primary education (*shōgakkō*), from the age of 6 to the age of 12, while secondary school (*chūgakkō*) is completed at the age of 15 and is the last compulsory stage. Practically all Japanese students continue their studies, entering high school (*kōkō*) from 15 until they are 18 years old. Finally, an entrance examination is necessary to pursue university (*daigaku*) studies.

The Ministry of Education, Culture, Sports, Science, and Technology (MEXT) is responsible for educational policies in Japan. The MEXT decides on the creation of new educational institutions and determines the budgets of each one of them, as well as subsidies granted to private centers. The Education Committee or the Governor of each prefecture establishes the amount of primary and secondary education centers needed to cover the demand.

The structure of the Japanese educational system is highly centralized and dominated by the national government [27], which unifies and standardizes education in a number of ways. It is the MEXT that publishes the guidelines for the national curricula (*gakushū shidō yōryō)* for primary and secondary education, as well as high school, determining both their shape and content. According to Sugimoto, “the Ministry of Education controls the content and tone of all school textbooks, supervises curricula throughout the nation, and has considerable power over the administration of universities”. As a matter of fact, its political and ideological stance, along with its concentration of power, has been at the heart of the debate.

In 1984, the Prime Minister created an ad hoc council for the reform of education, which is a work still in progress well into the 21st century. At the core of the reform is the change in the educational model: from memorization to the use of active learning methodologies that enhance the development of soft skills, such as critical thinking, tolerance, and creativity [28].

### 4.2. The Academic Debate Behind the Framework Laws and the Curriculum

The educational system is ruled by the *National Curriculum Guidelines* (*gakushū shidō yōryō*), which are revised every ten years and contain the following: the curriculum for each school level; the subjects that must be taught, and the number of specific hours that must be dedicated in each grade to each subject; the general objectives of each subject; and the objectives and contents of each school year. In other words, the guidelines establish the legal framework with the duties and responsibilities to maintain a prescribed level of education for primary, secondary, and higher education. Sugimoto [27] (p. 131) points out that even though educators have debated whether or not the guidelines are legally binding for individual teachers, de facto, the MEXT uses the guidelines to force them to comply with the educational framework.

Interestingly enough, while on the surface, the Japanese Government may seem to control the educational contents and teaching methods tightly, it only determines the teaching framework. It is the Local Education Councils that, especially after the reforms carried out since 1980, have acquired a greater sense of responsibility, autonomy, initiative, and vision, adjusting the contents of the textbooks and classroom activities. Textbook publishers produce books that conform to the national curriculum, and MEXT reviews and approves them; then, the local boards of education select the textbooks approved by the Ministry to be used in the schools.

The debate on Japanese education is defined through two fundamental trends. The first one advocates for a more relaxed education or an education with some freedom (*yutori kyōiku*), while the second tries to respond to international pressures in evaluations in subjects such as Mathematics or Science and promotes the so-called *Action Plan for the Improvement of the Academic Capacity.*

This debate is not new, since in the backdrop of the Japanese economic crisis that began at the end of the 1980s and continued in the 1990s, various leaders from different sectors demanded an education that both developed students’ skills and provided a solid academic education. At the same time, the objective was for students to acquire creative abilities (such as problem solving and critical thinking) and autonomy to further their education under the paradigm of lifelong learning. An added preoccupation was the concern about stress and anxiety produced by an educational system marked by excessive academic demand. These demands led to the revision of the new guidelines introduced in 2002, which developed a more powerful version of the *yutori kyōiku* to enhance students’ desire for learning and their satisfaction with what was learned.

The MEXT approved a series of reforms that spanned from primary education to the third year of secondary school. The most important measures were the decrease in the number of hours devoted to academic subjects, the creation of a course of “integrated studies”, and the overall reduction of the number of hours of classes, eliminating classes on Saturdays and establishing a five-day week like in businesses. The MEXT describes integrated studies as cross-curricular studies in which students carry out problem-solving activities, which help them to learn to identify and explore by themselves problems of society and life [29]. Each school decides which topics will be explored. The “integrated studies” course [30] was intended to give schools the freedom to create learning experiences outside the traditional boundaries of the curriculum. Schools and teachers are encouraged to use the curriculum creatively by offering an interdisciplinary and comprehensive learning experience.

The contents included under this modality are neither part of the university entrance examinations nor are they intended to provide very defined learning outcomes, covering topics such as the study of the natural environment, education for a global world, sustainability, the development of social skills, or resilience. Various activities are carried out, such as evacuation drills or how to use fire extinguishing equipment. Therefore, the emphasis on integrated studies is more significant in regions recently affected by natural disasters or in high-risk areas. Kagawa and Selby [31] mention the case of a primary school in Kochi prefecture that, using 50 class periods per year, conducts activities such as creating evacuation maps, puppet shows on disaster risk prevention for younger children, evacuation drills, and first aid skills practice during the integrated study period.

The national debate on education focused on acquiring academic knowledge while still providing greater freedom intensified even more from 2003 onwards because, after implementing the new plan, the performance of Japanese students in international comparative tests such as PISA or TIMMS began to decline. In 2007, in response to criticism, the *National Assessment of Academic Ability* was introduced, with the objective of assessing the students’ academic performance as a basis for the following reforms.

In 2009, the fundamental changes focused on the increase in the number of hours dedicated to academic subjects and the implementation of standardized national tests at the end of grades 6 and 9, aiming to detect specific subjects’ specific deficiencies. In addition, there was an increase in the number of teachers hired, especially in schools where there were academic and behavioral problems.

The results could be noticed in the 2012 tests, where Japanese students had the highest total PISA score among all OECD member states. Nevertheless, the gradual changes did not alter the essence of reforms that began in the 1980s, and the improved academic performance did not diminish the *yutori kyōiku* reforms and their benefits.

## 5. Fostering Resilience through Formal Education after the 2011 Triple Disaster

The Basic Law on Education, which was enacted in 1947, was revised in 2006 to update it, reflecting the changes in the socio-economic context (knowledge-based society, technological innovations, internationalization/globalization, population aging). Additionally, *Basic Plans for the Promotion of Education* were to be periodically formulated in order to set adequate policies and measures in line with the times. The first comprehensive governmental plan regarding education was formulated in 2008, and the last one, the third one, was formulated in 2018.

In the 2006 revised *Basic Law on Education* [32], a further step had been taken to integrate aspects related to resilience, by including among the objectives of education those related to the development of skills focused on personal and social development and the protection of the environment, which is in consonance with the goals in Article 1 that revolve around the concept of *chi toku tai* (intelligence, wisdom, and health), referring to creating a balance between body and mind, or, as the Latin adage goes, “mens sana in corpore sano”. In the MEXT’s own words, fostering solid academic abilities, richness in mind, and healthy bodies.

Within those objectives, the following can be highlighted for their close relationship to resilience: “developing individuals’ abilities, cultivating creativity, and fostering a spirit of autonomy and independence by respecting the value of the individual” (Article 2), and “the national and local governments shall encourage education that takes place within the community and society, in response to the demands of individuals and of the community and society as a whole” (Article 12) [32].

The Triple Disaster was a turning point for educational policies, owing to the adverse effects it had on the physical and psychological development of children. Not only did natural ecosystems suffer extensive damage, but the habitat and livelihoods in the areas devastated by the tsunami and affected by the nuclear accident did so as well. Consequently, people’s physical and emotional health was affected, as levels of stress and frustration increased. This and other factors were the triggers for the need to train intellectual, productive, creative, and emotionally healthy individuals. An objective that is intended to be achieved with the *New National Curriculum Standards* announced in 2017 and implemented between 2020 and 2022 [28] that includes (a) “active learning”, also referred to as “learning by doing”, that fosters independent individuals and creates interactive learning experiences; (b) New subjects such as scientific exploration, generic exploration, comprehensive public history, and comprehensive geography.

The Council for the Implementation of Educational Reconstruction, set up in 2013, identified the same issues that the ad hoc Council had identified 30 years before and proposed some educational reforms for 21st-century education to the Government. The 2013 Council program included reforms in the curricula, educational administrative systems, university entrance exams, and partnerships between schools and local communities, in the same pedagogical terms as the proposal of the 1984 Council. The educational perspective was intended to continue to enhance the ability to behave independently (*shutaiteki ni koudou suru taido*). In addition, emphasis was placed on developing a “culture of disaster preparedness” (*bosai bunka*) in the local community. The so-called “Kamaishi Miracle” was a key event that led to the debate concerning the traditional education approach mainly based on “how to evacuate”, which had proved to be inadequate [4]. The Kamaishi elementary school did not suffer any personal losses when it was hit by the 2011 tsunami, unlike other schools where students, teachers, and personnel did not evacuate on time. Kamaishi constitutes the example on which to benchmark Disaster Education [25], such as the development of life skills (such an “independent mindset”) through everyday school activities [33].

A mega-tsunami had been predicted for the area over the next three decades, according to a 2005 warning. At the request of the Kamaishi city Education Council, Toshitaka Katada, a Civil Engineering Professor at Gunma University and a Disaster Prevention Specialist, worked with local schools on disaster preparedness programs. As a result, Takada led the first session at Kamaishi Higashi Junior High School in 2005, and by 2008, a detailed training program focusing on the history of tsunamis in the area, local geology, and “survival training” had been created. Moreover, Katada’s training content was reinforced with content based on local wisdom, such as “tsunami tendenko”, a lesson inherited from elderly residents [34].

By 2011, the children had already received disaster risk education and training for at least 3 years, which was an experience that saved the lives of 3000 Kamaishi pupils who ran to the higher ground when the tsunami warning was issued on 11 March 2011, demonstrating that the ability to think by themselves and act proactively and independently was not only complementary to knowledge about disaster prevention but also relevant and a priority in saving lives. The students were able to think constructively and make decisions based on the changing circumstances. In brief, the students proactive thinking allowed them “to take appropriate evacuation actions based on their own judgment” [33].

In summary, in the Japanese educational system, the integration of information and knowledge on DRR in the school curriculum is tackled along two main lines of action: through the so-called “science of disaster” and through “life skills for disasters”. The inclusion of these contents in the curriculum is not a simple and automatic process but rather represents a challenge in the positioning of Disaster Education in the Japanese system (Table 3). The main limitations this approach encounters are the availability of sufficient teaching hours and the lack of structured and systematic content appropriate to each of the developmental stages [25,35].

Another drawback of this approach lies in the lack of transversality, as subjects are independent from one another. Critical voices argue that natural disasters have not been comprehensively addressed and have focused more on what to do before and after a disaster than, for example, training students in evacuation. The differences between the departments responsible for these activities in the various administrative districts of Japan cause these activities to be treated independently, and therefore, they are not always coordinated.

A valid alternative to solve this problem would be the inclusion of a work plan based on “infusion”, which some countries in Asia Pacific have already introduced [37]. In this approach, DRR-related content would be distributed throughout the curriculum through interconnected readings, activities, and problems. Its implementation requires a consultative and multi-stakeholder approach that begins before the curriculum adoption cycle, as it would include the development of the entire sequence of content until its implementation: from the skills required for DRR through to the design of adequate materials or teacher training. Consequently, a high-level political orientation and multi-stakeholder management are required.

### 5.1. Sensitive Changes to Integrated Courses of Study

The *Gakushū Shidō Yōryō* (National Curriculum) applied after 2011 is a response to modify the basis of the 2002 reforms but still maintain the reduction in teaching hours, as well as the integrated study courses, in a more concentrated way in relation to the previous decade [38].

Changes were introduced in the teaching methodology with a commitment to developing reasoning skills, the ability to understand concepts, and “experiential learning”. With these changes, the aim was for the student to develop critical thinking and the ability to solve problems that coping with novel or sudden situations, such as a disaster or an emergency, can pose. These guidelines included, for the first time, the concept of “zest for living”. This idea refers to providing a balanced education comprised of the three pillars of knowledge, morals, and physical strength to create healthy, well-rounded individuals with strong academic skills and moral values who will perform well in a changing society. Furthermore, special activities such as school events, also included in the curriculum, emphasize teamwork and cooperation.

Educational authorities also produced various supplementary educational materials on the March 2011 disaster used in integrated study periods. In addition, the content of subjects, especially social and natural sciences, included specific approaches to natural disasters. One year after the 2011 Great East Japan Earthquake and Tsunami, the MEXT published a “Guide to the Compilation of Disaster Prevention in Schools”. These recommendations encourage a “zest for living” (*ikiru chikara*) and were distributed to all schools in 2013. The version distributed is based on the concept that emerged after the Great Hanshin-Awaji Earthquake of 1995 and was introduced in the 1998 curricular guidelines, the year in which MEXT published “The Zest for Living: Education for disaster prevention” [39].

### 5.2. Some Examples of Educational Disaster Risk Reduction Tools

The Japanese DRR model is holistic as it includes not only the human systems (human behavior in case of evacuation) but also the natural systems, which were focused on the creation and publication of hazard maps with the identification of potentially dangerous areas and safe areas. These approaches have proved to be more effective in mutual coordination; therefore, tools have been created and deployed to visualize the interaction between them [40]. In the following lines, some relevant tools are introduced.

Firstly, a basic exercise consists in designing a *Nigechizu* (逃 げ 地 図) evacuation map. It is a map drawn by hand by the students with colored pencils and using ropes to measure in order to make it to scale. Roads and evacuation sites are drawn on the map, so that the users become aware and understand the situation and risks in their respective areas [41].

Secondly, the DIG (Disaster Imagination Game) program was developed in 1997, based on the know-how of the Commanding Post Exercises of the JSDF [41,42]. This program uses transparent overlay maps showing essential facilities such as schools and hospitals, roads, rivers, and higher ground. Potential damages are also flagged as possible road disruptions. With all these signs, the evacuation map is built.

Finally, the Nige-Tore Program is an application for smartphones useful in case of tsunami. Compared to traditional tools that are static showing repeatedly the same escape route, Nige-Tore has the advantage of taking into account the variability of the real risks in a dynamic way. This program follows these steps: (1) Configuration and conformation of the hazard map. It shows the location of the person in real time, the potential danger zones, and the safe places (high places, high evacuation buildings, or other places designated by the municipal authorities). (2) Action start. The system allows the user to control the relationship between his/her position in real time and the advance of the tsunami. (3) Assessment and reflection. The user’s success level appears on the screen, informing whether he or she has been more or less close to the danger zones [40]. As in the case of Kamaishi, the need for the subject to make his/her own decisions and act following his/her own judgment based on the specific behavior of the catastrophe is at the heart of the application.

## 6. Discussion

Various lessons can be extracted from this research, lessons from the Japanese educational system that can be put into practice elsewhere.

### 6.1. Formal, Non-Formal, and Informal Education

UNESCO’s Unevoc compiles a glossary of terms in which formal, non-formal, and informal education, together with the definitions of multiple other terms, are recorded. In that glossary, formal education is defined as the kind of “education provided in educational institutions, such as schools, universities, colleges, involving direction from a teacher, and constitutes a continuous ladder of full-time education”. On the other hand, informal education is “institutionalized, intentional and planned by an education provider, but it is an addition, alternative and a complement to formal education”. Finally, informal education and training is “unstructured education/training that takes place outside the formal education/training system”, such as “learning resulting from daily life activities related to work, family or leisure”.

DRE programs should combine the three kinds of education in order to generate significant learning, i.e., receivers can relate new knowledge to previous knowledge so that both old and new knowledge get mutually reinforced, and this makes it easier to remember the information when needed (Table 4).

As mentioned above, in Japanese schools, disaster preparedness is inserted in the study programs and taught as part of the curriculum in basic subjects, both in formal (for example, in natural sciences, students could learn about weather events, the contents would be in their textbooks), non-formal (in integrated learning, for example, with specific training and education on DRR) and informal contexts (a school festival).

It is necessary to define which contents will be taught and how those contents will be administered. Disaster risk preparedness is an interdisciplinary field; therefore, it can be included in various formal subjects, making it an excellent topic for various other non-formal projects.

### 6.2. Combine Theory and Practice in “Everyday-Life Disaster Preparedness” (Seikatsu Bosai)

All school-age children in Japan, from kindergarten to high schools, receive regular training and courses on risk management. The Miracle of Kamaishi is the best example of how drills regularly practiced can be reproduced, if or when the need arises so that lives can be saved. The pupils of Kamaishi Junior High School evacuated to higher ground on 11 March 2011, when the tsunami alert was issued. Acquiring skills through practice is essential for students to be able to act proactively when an emergency arises. Regular drills practicing emergency evacuations, first aid techniques, and other practical skills should be included in school curricula.

“Disaster Prevention Day” or *bosai no hi* is celebrated in Japan every year on 1 September, on the anniversary of the 1923 Great Kanto Earthquake (also known as the Tokyo Earthquake), which killed 100,000 people. It is a day in which various disaster-related activities are held all over Japan, such as seminars, exhibitions, or evacuation drills, amongst others. Celebrating a Disaster Prevention Day locally, even if it is not in the Japanese date, can be a way of raising awareness and keeping survival skills sharp.

### 6.3. Make Children DRR Ambassadors

Children can be the ones teaching other family members (parents, grandparents, younger siblings) by transmitting the information they have learned at school. As receivers of a series of teachings that have been standardized around the country, Japanese children bear witness to the lessons learned by past generations and will pass them on as adults. Children are the future; therefore, it is essential to educate them in DRR so that today’s children become responsible citizens in the future.

### 6.4. Generate Community Involvement

As elsewhere, Japan is experiencing an inevitable decline in community life as people become more individualistic and stay at home instead of participating in community activities. This is especially alarming in big cities, where community life is reaching an all-time low in some neighborhoods.

In the event of an emergency, it is essential not to leave anyone behind. A tightly knit and well-networked community can support everyone, especially those with special needs, such as the elder or people with disabilities. Community activities such as festivals, fire drills, and other disaster-related activities can contribute to creating community.

### 6.5. Adopt an All-of-Society Approach

Young and old, men and women, Japanese and foreigners, citizens, businesses, charities, NGOs, local governments: it is essential to involve everyone because disaster risk reduction is something that concerns everyone.

## 7. Conclusions

Due to its location in the Pacific Ring of Fire, Japan is a country prone to disasters, which has led it to adopt a proactive stance. The experience acquired throughout the years in disaster management has made it an international referent in disaster risk reduction. Japan’s good results in crisis management highlight the importance of creating resilient communities to face potential risks.

As they grow in size and population, the challenges of urban areas will be exacerbated by risk drivers such as climate change, weak governance, economic and social inequalities, or health issues, among others. Building resilience into cities can positively contribute to improving the lives and livelihoods of city dwellers, whatever their socio-economic status might be.

In this article, it has been contended that education is a powerful tool to foster resilience in children, teenagers, and young people. However, in order to achieve this goal, a positive change is necessary. Such change, the article has contended, should be carried out from a double perspective: top–down and bottom–up.

From a bottom–up perspective, policymakers should consider introducing in the curricula DRE, either as a separate subject or as part of existing ones. Furthermore, the need for active learning and soft skills development should take preeminence over memorizing facts and repeating exercises. The year 2011 was a turning point for Japan in many aspects: economic, political, and social ones, but also in education. Japanese education is said to be both first-class and uncreative. The Triple Disaster brought about some educational reforms seeking to foster soft skills such as tolerance to incertitude, critical thinking, problem-solving, and creativity, without losing sight of the hard skills. The *New National Curriculum Standards*, in place since 2020, seek to foster intellectual achievement and educate balanced and emotionally intelligent individuals.

From a bottom–up perspective, this formal school education is complemented by informal learning and training. In Japan, there are three levels of support: government support, community support, and self-help. Community support is the domain of an active civil society involved in various disaster prevention activities, such as cooperating with schools in various activities to raise awareness: festivals, seminars, and fire drills. There are voluntary disaster prevention organizations (*jishu bosai soshiki*) in every neighborhood, as well as fire control clubs (*bosai kurabu*), volunteer civil society organizations that work on disaster preparedness.

The ability to be prepared for unforeseen events has proved to be a fundamental skill throughout human history. Nevertheless, recent events such as the COVID-19 pandemic have placed this ability at the center of the educational debate. As key community agents, schools must consider disaster risk reduction from a holistic perspective that integrates not only disaster response but also disaster prevention and risk management. Both formal and informal educational agents share the responsibility of teaching and training.

Social networks are an essential part of Japanese culture, which is group oriented. Therefore, participating in DRR community activities is a double opportunity to foster resilience: by training one’s *seikatsu bosai* (everyday life disaster preparedness) and by strengthening community ties. Human relationships are essential not only in the moment when a disaster strikes (young people helping older mobility-challenged people so that no one is left behind), but also in post-disaster scenarios where psychological support helps make sense of life.

The cases analyzed in this article and the lessons extracted might prove helpful for educators, policymakers, and researchers who seek points of action, from the educational sphere, in the area of Disaster Risk Reduction. As a result of the COVID-19 pandemic, the current situation of crisis should also be taken as an opportunity to advance educational policies that develop curricular content that addresses disaster risk reduction and soft skills. From a systemic approach, schools are in continuous connection with individuals and their immediate environment (family and neighbors), as well as with communities and the society at large. Consequently, in a system characterized by mutual feedback and the generation of synergies, to create more resilient societies and cities, it is first necessary to address educational systems’ challenges.

## Figures and Tables

**Table 1:** Seven approaches to urban resilience.

Urban Resilience Core Concept	Practical Application	Differentiating Features
Disaster resilience	*UNDRR*	Surviving high-impact low-frequency events and getting basic urban functions back quickly
Engineering resilience	*UN-Habitat*	Withstand and recover quickly from hazards (bounce back)
Ecological resilience		Speedy recovery to a state of equilibrium. Having a functioning system
Socioecological resilience	*Resilience Alliance*	Holling’s original definition: a system’s ability to absorb disturbances while remaining within an acceptable state. Extends ecological resilience to include human and cultural elements.
Evolutionary resilience	*City Resilience Index*	Cities are dynamically changing systems. Four phases: growth, conservation, creative destruction, and reorganization. Recovery as an opportunity to build back better (bounce forward)
Built-in-resilience	*Resilience Rotterdam*	Capability in physical, institutional, economic, and social terms to keep adapting to existing and emergent threats
Climate change resilience	*Australia’s National Climate Resilience, and Adaptation Strategy*	Quickly bounce back from climate-related shock and stresses. Focus on climate change mitigation and adaptation
Other conceptualizations		Stable and unstable resilience. Anticipatory and reactive resilience. General resilience

Source: Elaborated by the authors based on Sanchez, van der Heijden, and Osmond.

**Table 2:** Summary of the contributions of the UN World Conferences on Disaster Risk Reduction (WCDRR).

WCDRR	Venue (Year)	Document	Time Frame	Contributions
3rd	Sendai (2015)	*Sendai* *Framework for Disaster Risk* *Reduction*	2015–2030	Invest in DRR to build resilience. Strengthening the governance of organizations for DRR at the national level. Coordinate the efforts of the public and private sectors, as well as civil society. Learn from disasters and “build back better” (BBB)
2nd	Kobe, Hyogo Pref. (2005)	*Hyogo* *Framework for Action*	2005–2015	Mainstreamed the concept of resilience. Improve community resilience by strengthening and training the community. Emphasis on education on disaster risk and safety in school facilities.
1st	Yokohama (1994)	*Yokohama* *Strategy and Plan of Action for a Safer World*	1994–2005	Guidelines that should guide risk prevention, preparedness, and mitigation. Paradigms change for the UN, from Relief to Reduction (from post-disaster relief to prevention). The UNDRR is born (successor of UNISDR)

Source: Elaborated by the authors.

**Table 3:** Synthesis of sample activities for disaster risk reduction in the school curriculum, according to the MEXT.

Stage	Example of Curriculum
Elementary School (age: 7–12)	Life (2nd grade): Public facilities and workers, for example, fire station and firefighters Science (5th grade): How running waterworks Science (6th grade): Earth changes driven by volcanic eruptions, earthquakes, and floods Health and physical education (5th grade): Injury prevention Home economics (5th and 6th grade): Outdoor cooking exercise
Lower Secondary School (age: 13–15)	Science: Characteristics of volcanic eruptions and earthquakes Health and physical education: Injury prevention and first aid in times of disaster Home economics: Outdoor cooking exercise
Upper Secondary School (age: 16–18)	Health and physical education: First aid training in a time of disaster Ethics: Dignity for life, Relationship with nature and science Science: Mechanism of volcanic eruptions and earthquakes Home economics: Cooking and living in times of disaster
Besides the above, drills for evacuation in case of disaster, lectures on disaster prevention by experts, science club activities, volunteer activities in times of disaster, and others, are implemented as class activities, school events, and club activities.	

Source: Nakamura [36].

**Table 4:** Disaster Resilience Education programs in Japan.

	Formal Learning	Non-Formal Learning	Informal Learning
**Name**	School subjects in the curriculum	Integrated learning	Community event
		One-off type	
**Examples**	Natural sciences	Stand-alone activities such as “bosai no hi”	a festival a sports event (*undōkai*)
**Objective**	Educate	Raise awareness, design teaching materials	Entertain and inform
**Stakeholders**	Schools/students	Schools/students	Schools/students, households, communities

Source: Elaborated by the authors.

## Data Availability

The data that support the findings of this study are available from the corresponding author, upon reasonable request.
